# Parylene C as a versatile dielectric material for organic field-effect transistors

**DOI:** 10.3762/bjnano.8.155

**Published:** 2017-07-28

**Authors:** Tomasz Marszalek, Maciej Gazicki-Lipman, Jacek Ulanski

**Affiliations:** 1 Organisch-Chemisches Institut, Ruprecht-Karls-Universität Heidelberg, 69120, Heidelberg, Germany; 2 InnovationLab, Speyererstr. 4, 69115 Heidelberg, Germany; 3 Department of Molecular Physics, Lodz University of Technology, Zeromskiego 116, 90-924 Lodz, Poland; 4 Institute for Materials Science and Engineering, Lodz University of Technology, Stefanowskiego 1-15, 90-924 Lodz, Poland

**Keywords:** dielectric, encapsulation layer, flexible substrate, organic field effect transistor, Parylene C

## Abstract

An emerging new technology, organic electronics, is approaching the stage of large-scale industrial application. This is due to a remarkable progress in synthesis of a variety of organic semiconductors, allowing one to design and to fabricate, so far on a laboratory scale, different organic electronic devices of satisfactory performance. However, a complete technology requires upgrading of fabrication procedures of all elements of electronic devices and circuits, which not only comprise active layers, but also electrodes, dielectrics, insulators, substrates and protecting/encapsulating coatings. In this review, poly(chloro-*para*-xylylene) known as Parylene C, which appears to become a versatile supporting material especially suitable for applications in flexible organic electronics, is presented. A synthesis and basic properties of Parylene C are described, followed by several examples of use of parylenes as substrates, dielectrics, insulators, or protecting materials in the construction of organic field-effect transistors.

## Review

### Introduction

An improvement of the performance of organic transistors by means of boosting charge-carrier mobility is one of the main quests in organic electronics, calling for novel design of molecular materials and enhanced processing conditions. Over the past 20 years, the work has been mainly dedicated to the selection and processing of organic semiconductors: either small molecules [1–2] or systems with high molecular weight [3–4]. Nevertheless, it should be pointed out that it is not only semiconductors that constitute crucial elements of organic field-effect transistor (OFET) architecture. The role of both interfaces, namely those of dielectric/semiconductor [5–7] and semiconductor/electrode [8–9] is widely discussed in the literature. In addition, elements such as electrodes [8], substrate [10] and protective layer [11] are considered to have a significant impact on the transistor performance as well. A particularly important role in the field-effect response is supposed to be played by a dielectric material, a notion that has been accentuated by several reports [12–13]. When this type of material is considered for an application in organic transistors, specific requirements for the gate insulator have to be fulfilled. The most important of these requirements comprise high capacitance, substantial dielectric strength, high purity and processability of the material. In addition the material should yield device characteristics such as high on/off ratio, low hysteresis, and long-term stability. There are only few reports that describe, in a comprehensive way, an influence of each element on the performance of the organic transistor [12,14–15].

At present, one of the most important utility features in the field of potential organic-transistor applications is the flexibility of the semiconductor layer deposited on top of a polymer substrate [16]. In the best case, no degradation of device performance was observed for bending radii as small as ca. 200 μm [17]. Measured variations of the charge-carrier mobility [18] were assigned either to mechanical changes in the semiconductor film or to charge trapping at the dielectric/semiconductor and semiconductor/electrode interfaces. It should be pointed out that the primary element affecting the transistor flexibility is a substrate that is not only flexible (relatively low Young's modulus) but also offers a smooth and pinhole-free structure. An equally important role is played by the encapsulation layer. Firstly, it protects the semiconductor thin film against the negative influence of water and oxygen. Secondly, it makes the semiconductor thin film remain in its initial position during the bending process, which prevents a charge trapping effect induced by the mechanical cracking [19]. For this reason, there is substantial interest in polymer materials that can be successfully applied in flexible organic transistors as both substrate and encapsulation layer.

The present work is focused on the unique performance of one polymer material used in OFETs. This material is poly(chloro-*p*-xylylene) (Parylene C) the applicability of which in the field of OFET manufacturing appears to be continuously growing. Three properties of Parylene C, treated here as independent application fields, are found useful in a fabrication of high performance organic transistors. First of all, major advantages of the chemical structure and the deposition procedure of this polymer are pointed out with the focus on its application as a flexible substrate. Secondly, the electrical insulating properties of this material are presented with emphasis on its use as a gate dielectric material. Last, but not least, an advantage of encapsulation properties of Parylene C, earlier applied in the area of conservation [20–22] are currently utilized in a form of protective layers stabilizing organic electronic devices. It should be pointed out that the requirements for the barrier protecting an organic transistor (about 10^−2^ g/m^2^ per day) are not as restricted as those regarding an organic photovoltaic (OPV) device (10^−4^ g/m^2^ per day) or an organic light-emitting diode (OLED) (10^−6^ g/m^2^ per day) [23]. Nevertheless, some of the organic semiconductors (mostly electron-transporting materials) require an encapsulation layer, in order to observe charge transport in the transistor architecture [24]. According to our knowledge, there is a limited number of materials that can be simultaneously used as a substrate, dielectric and encapsulation layer at the same time while presenting a performance comparable to the materials dedicated to the specific application [25].

### Synthesis of Parylene C

**Figure 1:**
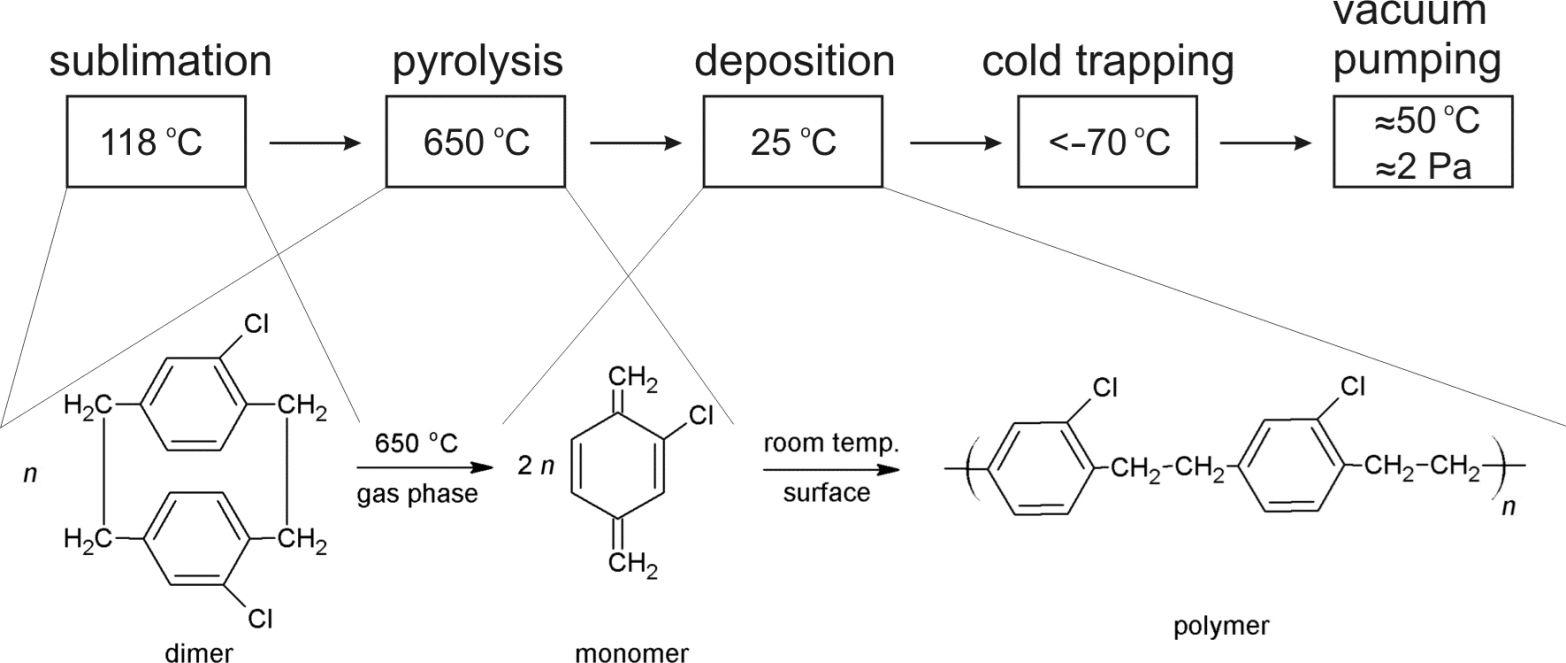
Schematic representation of the deposition process of Parylene C with the respective chemical reactions. Reprinted with permission from [28], copyright 2016 Elsevier.

The process of deposition of xylylene polymers, known under the commercial name of parylenes, is unique in many ways. It is a synthetic path for polymer formation, at the same time it belongs to the category of chemical vapor deposition (CVD) and, as such, it yields products in a form of conformal solid films depositing at any surface exposed. As a CVD process, on the other hand, it results in the formation of organic polymers with high molecular weight, whereas typical products of these processes are inorganic materials of either metallic or ceramic nature. Perhaps the most unusual feature of the parylene process is the polymerization mechanism itself. The initiation step does not require any external initiator but, instead, it involves a monomer molecule in its diradical triplet first excited state [26]. A natural consequence of this mechanism is the extraordinary purity of parylene coatings, a property of great importance in electronic applications. Yasuda et al. [27] pointed out first that this purity results in a low concentration of localized states at the dielectric/semiconductor interface of the OFET. The authors investigated a number of poly(*para*-xylylene) derivatives with regarding their effectiveness as gate dielectric layers in OFET devices. In each case, independent of the active material used, out of six different xylylene polymers the highest field-effect mobility was exhibited by the transistors equipped with a Parylene C dielectric layer [27]. A schematic diagram, showing the stages of the Parylene C deposition process, together with the accompanying chemical reactions, is presented in Figure 1.

There is a number of advantages of the parylene technology. First of all, being a gas-phase diffusion-controlled process, it yields smooth pinhole-free conformal coatings with excellent penetration abilities. Second, there are several benefits resulting from the fact that the deposition takes place at or around room temperature. The two most important ones are the capability to coat thermolabile substrates [20–22] and the avoidance of mechanical stress otherwise introduced by different thermal expansion coefficients of coating and substrate. Finally, as it has been already stressed above, the polymerization reaction is initiated spontaneously and as such it requires no external initiator/catalyst. This unique feature makes the product uncontaminated with impurities influencing electrical conduction. As far as the termination of the reaction is concerned, there is none as long as the growing macromolecules remain under vacuum. The polymerization reaction exhibits a step-growth mechanism with second order kinetics with respect to the active radical sites [26]. Upon exposure to the atmosphere, these radical active centers (sometimes described as “dangling bonds”) are quenched with oxygen, forming oxide-type moieties [26]. However, because the gas permeability of parylene coatings is low and the degree of polymerization is very high [26], the concentration of these structures and, therefore, their effect on electrical conduction of the polymer is low.

**Figure 2:**
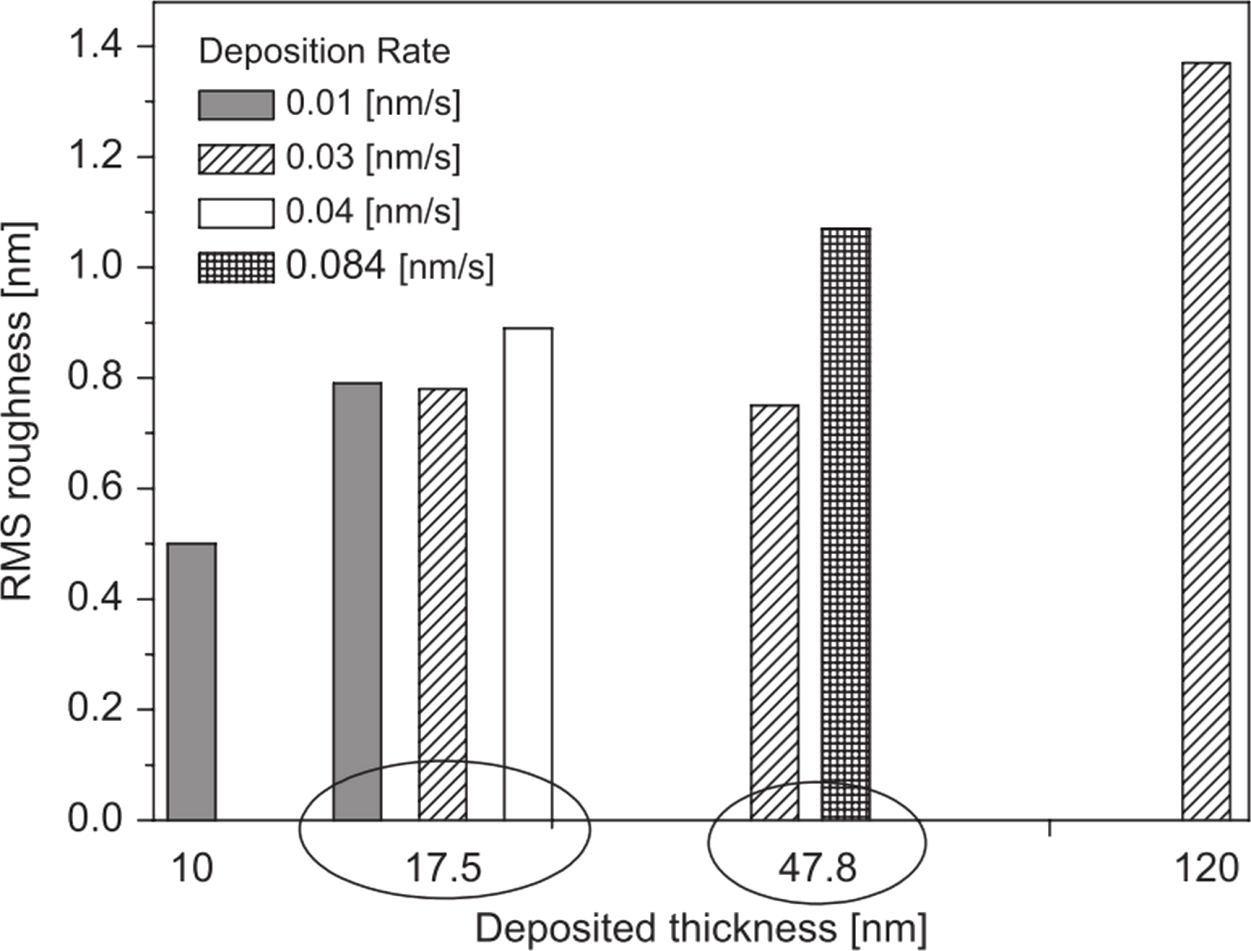
AFM measurements of the surface roughness of Parylene C thin films. Reprinted with permission from [30], copyright 2009 Elsevier.

It is known that Parylene C films deposited at high pressure and high deposition rate are rough and have non-uniform and poor dielectric properties. A small increase of the deposition rate from 0.015 to 0.08 g/min results in a growth of the root-mean-square surface roughness from 5.78 to 9.53 nm [29]. The same effect of an increasing roughness with increasing deposition rate was observed when various film thicknesses were compared (Figure 2) [30]. Therefore, when increasing the sublimation rate, one should be aware of the resulting increase of the film surface roughness.

**Figure 3:**
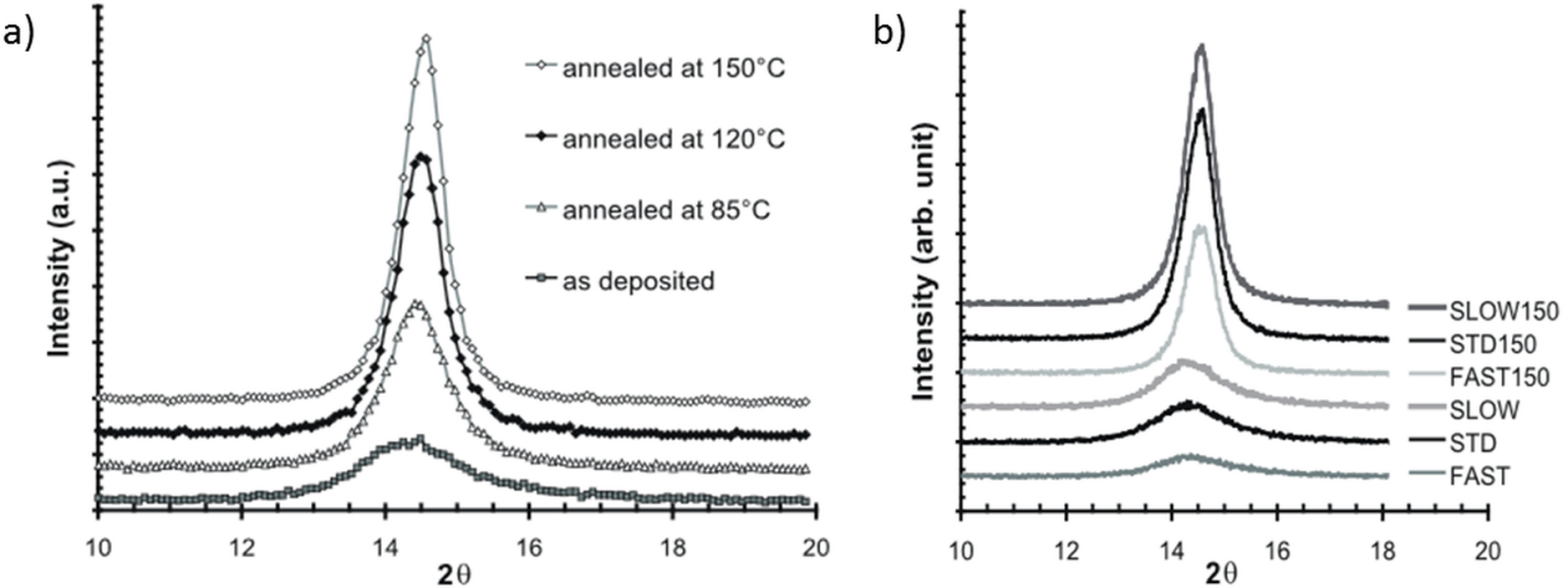
XRD spectra of Parylene C films: as-deposited with constant deposition rate and thermally annealed at different temperatures (a), as-deposited with different deposition rates and thermally annealed at constant temperature (b). Reprinted with permission from [29] copyright 2008 MYU K.K. (reprinted from electronic version).

Xylylene polymers are partially crystalline materials. It was found that both deposition rate and post-deposition thermal treatment significantly affected the crystallinity of the Parylene C films. Both as-deposited and thermally annealed films were subjected to X-ray diffraction (XRD) measurements and showed a maximum at 2θ ≈ 14.5° corresponding to the (020) crystalline plane (Figure 3) [29]. It can be seen that the peak height increases with the annealing temperature, while the full width at half maximum (FWHM) is observed to decrease.

The interlayer distance (*d*-spacing), which decreases with increasing temperature, indicates that more ordered polymer chains are formed at higher temperatures. This is due to higher energy available for chain motion and crystallization during thermal annealing. The size of crystalline domains is controlled by a number of defect mechanisms in the polymerization process. The crystallinity of Parylene C films affects their mechanical properties such as elastic modulus and/or Poisson’s ratio. The sample with higher crystallinity has approximately 30% greater tensile strength than the as-deposited films, a feature highly required from the point of view of material flexibility. The high quality of Parylene C thin films was confirmed by micro-Raman spectroscopy. The principal Raman band localized at 1336 cm^−1^ was assigned to C–H in-plane deformation in accordance with the results obtained earlier for both a bulk Parylene crystal [31] and micrometer thick layers [32]. Good surface homogeneity in the micrometer range [30] was revealed by means of mapping the layers with micro-Raman spectroscopy, where only small differences in Raman intensity in all measured positions were observed.

### Parylene C as substrate material

**Figure 4:**
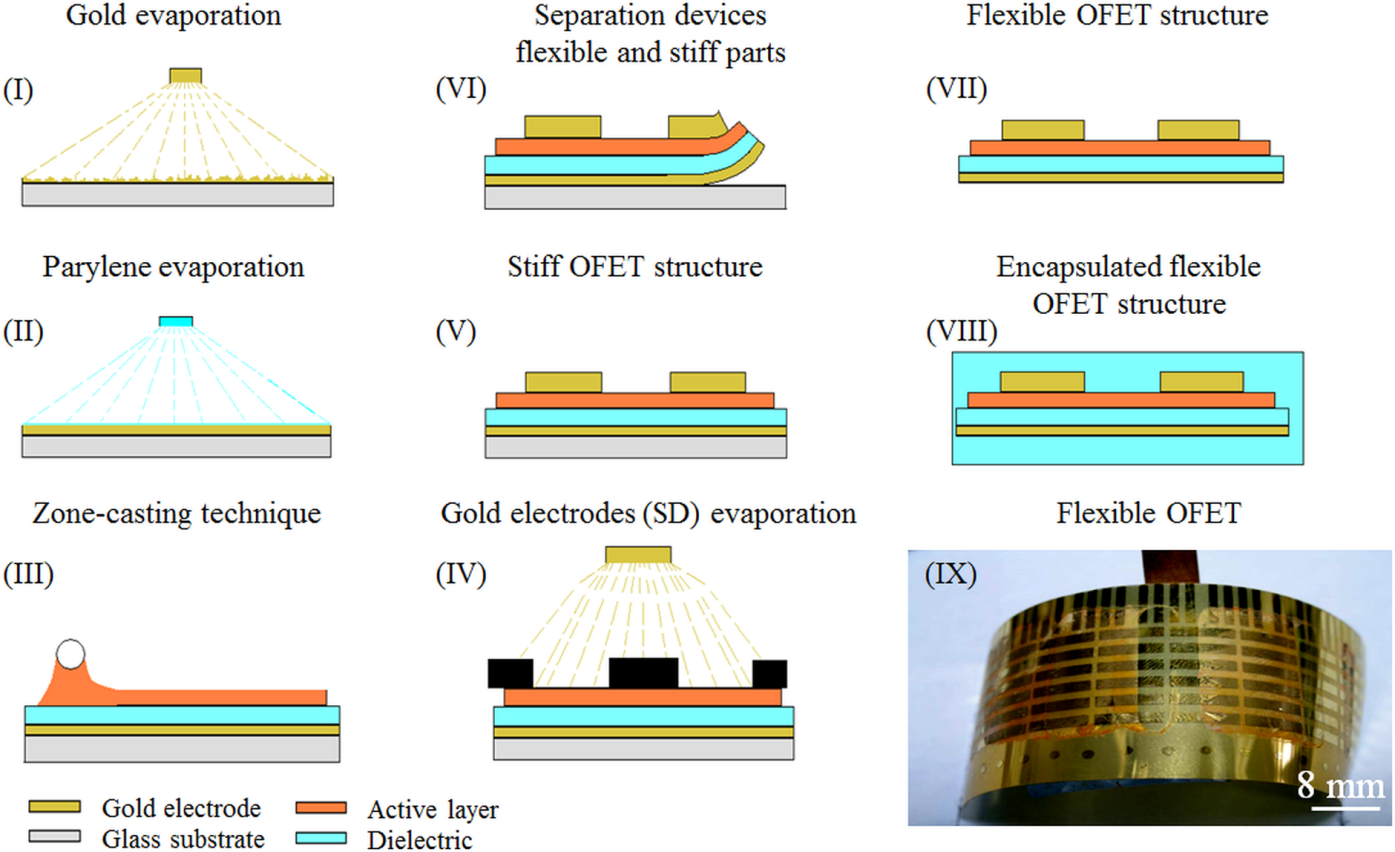
Schematic illustration of the flexible OFET fabrication procedure with Parylene C as a substrate and gate dielectric layer and with zone-cast tetrakis(alkylthio)tetrathiafulvalene as semiconductor. Reprinted with permission from [34].

In the transistor configuration presented in Figure 4, Parylene C is not only used as a gate dielectric material but it also serves as device flexible substrate. Such a flexible substrate allows one to investigate the influence of mechanical bending on charge carrier transport in the zone-cast layer of tetrakis(alkylthio)tetrathiafulvalene [18]. Bending tests carried out for numerous curvature radii clearly demonstrate that the performance of OFET devices (with structure presented in Figure 4(VII)) does not deteriorate irreversibly under these conditions. When subjected to bending, the devices still work, with the calculated mobility gradually dropping off with a decrease of bending radius. While amounting to 0.1 cm^2^/Vs for unbent structures, its magnitude decreases to ca. 0.06 cm^2^/Vs for *r* = 25 mm and to 0.04 cm^2^/Vs for *r* = 5 mm [18]. This effect has been attributed to the influence of stress induced in the tetrathiafulvalene (TTF) semiconductor crystalline films, namely charge trapping caused by mechanical bending [33].

**Figure 5:**
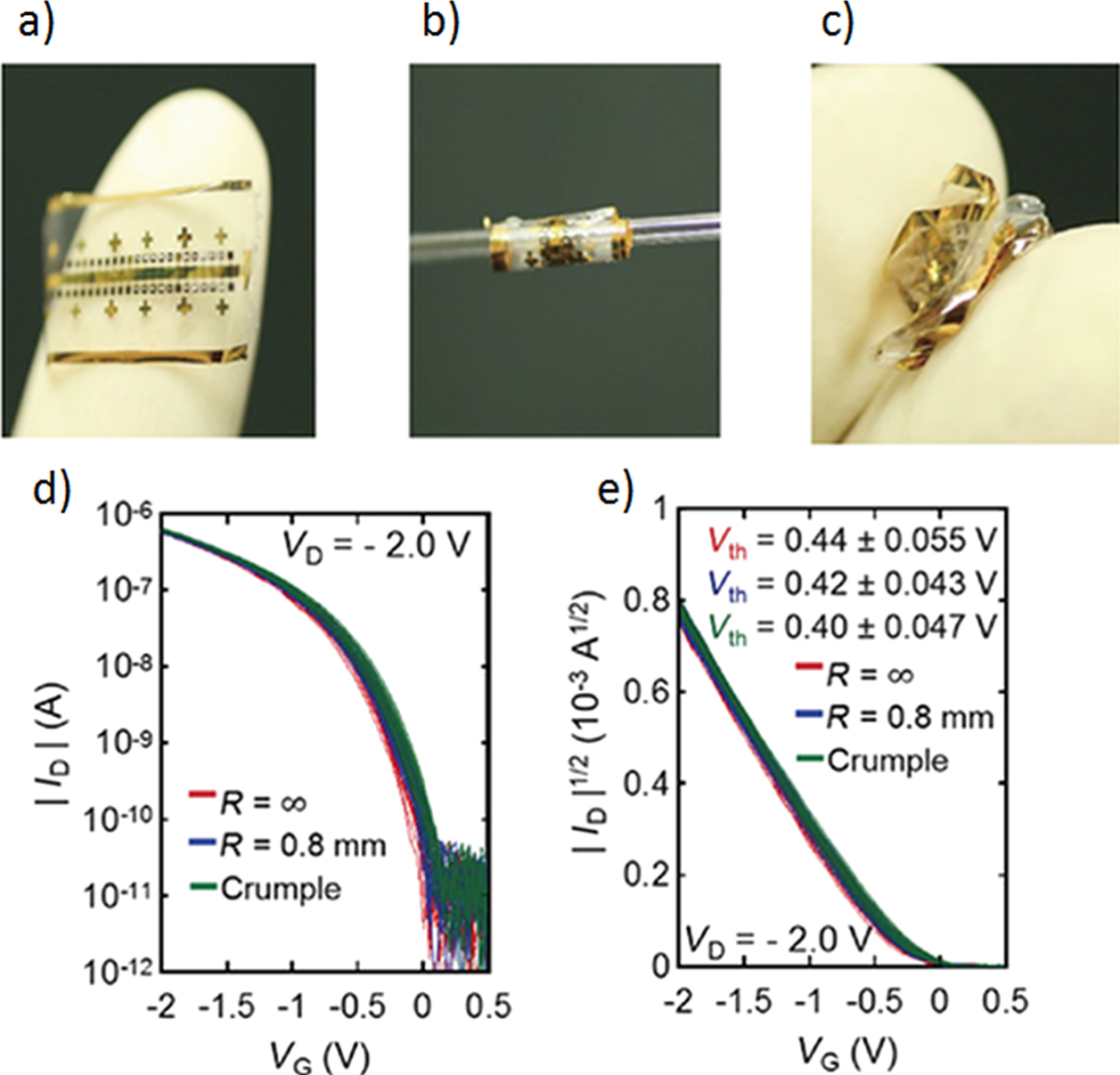
Transfer characteristics of 10 OTFTs after bending and crumpling tests: (a) Photograph of a device before mechanical tests. (b) Photograph of a device rolled onto a cylinder of 0.8 mm radius. (c) Photograph of a crumpled device. (d) and (e) Transfer characteristics of 10 OTFTs before and after bending and crumpling tests. Reprinted with permission from [33] copyright 2016 IOP Publishing Ltd.

In another work, ultra-thin Parylene C insulating layers were fabricated on Au gate electrodes by reducing the parylene film thickness to 18 nm with the help of oxygen plasma etching [33]. This procedure enabled the manufacturing of OFET devices with a driving voltage as low as 2 V. In fact, the OFETs equipped with the 18 nm thick parylene gate insulator exhibit excellent low gate leakage currents (of the order of picoamperes and below) at 2 V operation. Mechanical tests of OFETs fabricated on a 3 μm thick Parylene C film were carried out. However, the main difference between the results obtained for TTF derivatives, described above, and those obtained in [33] is that the transistor was additionally encapsulated with 3 μm thick Parylene C coating to set it on a strain neutral position. Figure 5a presents a device bent onto a 0.8 mm radius glass tube in the course of a bending test. Transfer characteristics of ten OFET transistors collected before and after the tests are presented in Figure 5d [33].

As seen in Figure 5, OFET transfer characteristics show a narrow dispersion and a gate leakage current of the order of picoamperes, and these properties do not change after mechanical tests. The remaining transistor parameters such as charge carrier mobility, subthreshold and threshold voltage also remain practically unaffected by mechanical testing. The threshold voltage value, 0.44 V for the unbent device, became slightly reduced down to 0.42 V and 0.40 V after bending and crumpling tests (Figure 5e). The results show that OFET devices with the 18 nm thick parylene gate insulator are characterized by a similar mechanical durability as those equipped with a 100 nm thick layer of the gate insulator [35]. It could be concluded, on a basis of the results obtained for bent transistors, that the encapsulation layer substantially improves mechanical properties of the devices.

### Parylene C as a gate insulating layer

**Figure 6:**
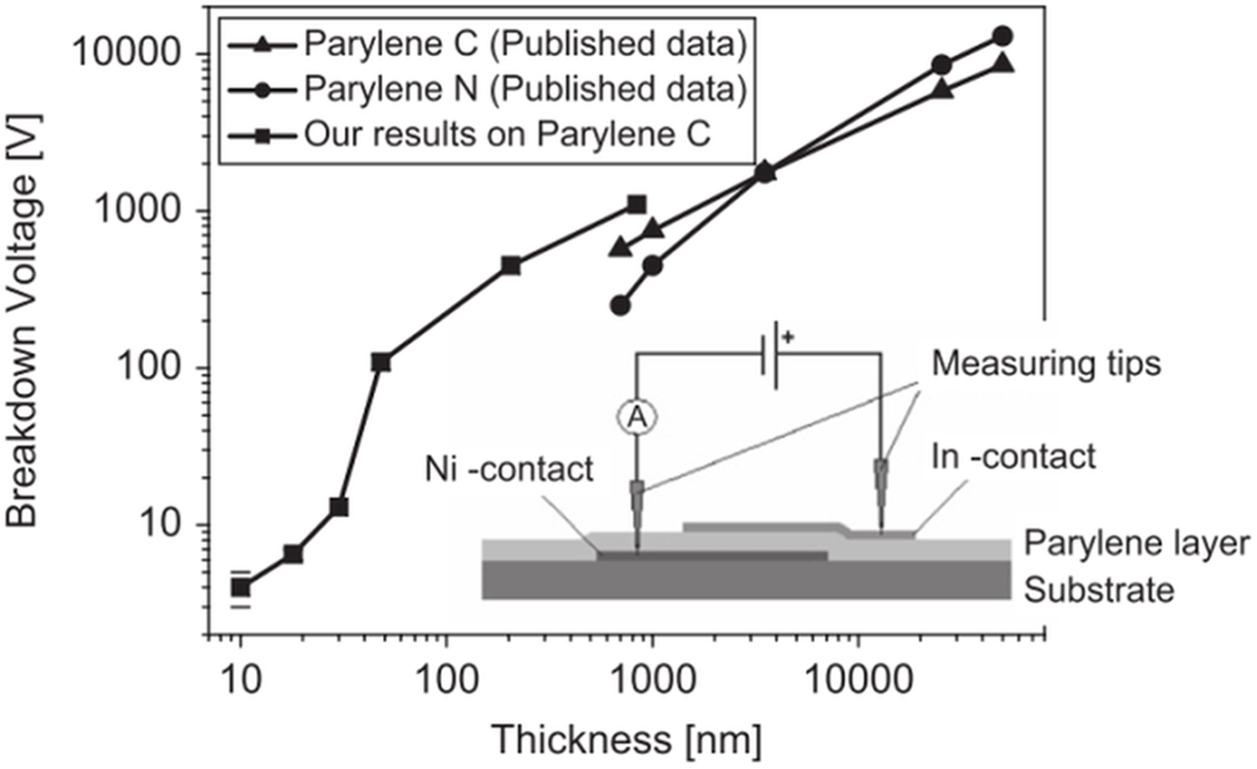
Thin Parylene C layers breakdown voltage as a function of thickness. Reprinted with permission from [30], copyright 2009 Elsevier.

The purity of thin dielectric films has a tremendous impact on their electrical properties. Results of electrical breakdown voltage measurements on a 2 mm × 2 mm area capacitor structures equipped with a dielectric layer of Parylene C are presented in Figure 6.

The measurements in Figure 6 reveal the excellent electrical properties of Parylene C layers, particularly in terms of their dielectric strength. An additional advantage of these films is that the breakdown voltage remains constant across the entire capacitor area, which is typical for uniform and pinhole-free layers. The above results show that thin Parylene C films are good candidates for the gate insulating material in organic thin film transistors.

**Figure 7:**
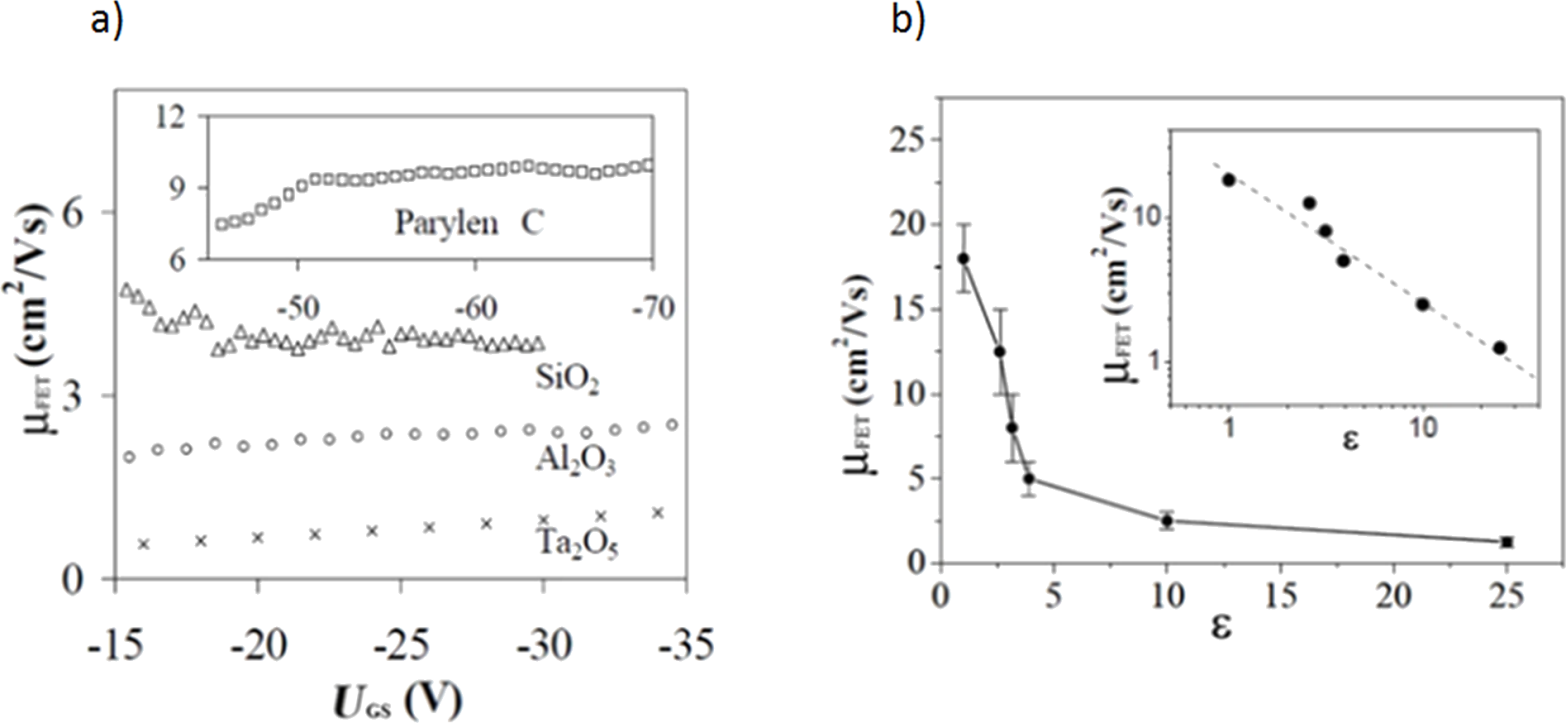
(a) Mobility μ(*V*_g_) curves measured for four different gate insulators. For the device based on Parylene C, the suppression of contact effects often requires a rather large value of *V*_DS_ (and thus *V*_GS_) to remain in the linear regime. (b) Decrease of the mobility with increasing ε, as observed in rubrene single-crystal FETs with different gate insulators. The bars give a measure of the spread in mobility values. Inset: when plotted on a log–log scale, the available data show a linear dependence with slope −1 (i.e., the variation in μ is proportional to ε^−1^). Reprinted with permission from [37], copyright 2004 of AIP Publishing.

For an application, it is required that a transistor has to be controlled by the lowest possible voltage. A thickness decrease of the dielectric layer allows one to reduce the applied gate voltage, with a drawback being an increased leakage current [36]. The efficiency of the field effect is dependent on the capacitance of the gate insulating material. The capacitance is determined by the dielectric permittivity (ε) and the thickness of the insulating layer. Currently, two types of dielectric materials are commonly employed in transistor design and construction, either inorganic metal oxides (such as Ta_2_O_5_, Al_2_O_3_, SiO_2_) or organic polymers [13]. However, it was found that the application of an inorganic insulator with high ε significantly decreases the mobility of charge carriers by interaction with the induced polarization in the gate insulator [37]. The effect of dielectric permittivity of the gate insulating material on field-effect mobility, investigated in rubrene single-crystal transistors equipped with various dielectrics layers, is shown in Figure 7. In Figure 7a, for the device based on Parylene C, the suppression of contact effects requires a larger *V*_DS_ value (and thus also a larger value of *V*_GS_), in order to remain in the linear regime.

To summarize, it should be pointed out that an increase of dielectric permittivity of gate insulating material results in a decrease of field effect mobility (Figure 7b). For all dielectric materials applied, the highest values of charge carrier mobility were obtained for xylylene polymers. In the case of Parylene C (ε = 3.15) it was approximately 10 cm^2^/V·s, while for Parylene N (ε = 2.65) it varied in the range of 10–15 cm^2^/V·s. In contrast, an application of the oxide gate dielectric Ta_2_O_5_ (ε = 25) resulted in a lower mobility value of 1.5 cm^2^/V·s [37]. The maximum value of 16–20 cm^2^/V·s, was obtained for vacuum as a dielectric [38].

The deterioration of the dielectric/semiconductor interface was revealed to be due to charge trapping at that interface. This effect could be controlled by an application of self-assembly monolayers (SAM) that significantly reduce the number of traps but they cannot entirely eliminate surface SiOH groups [39]. When polyethylene was used as a buffer dielectric, unhindered charge transport was observed [40], suggesting that thin polymer layers could play the same role as SAMs do. For this reason, polymers are often used as a part of twin dielectric layer systems in which one layer is responsible for dielectric strength and/or capacitance, whereas the other is designed to form a preferred interface for the growth of an organic semiconductor [41]. Due to a substantial charge-trapping effect observed in inorganic dielectrics, the organic polymer insulators bear much higher application potential in organic transistor technology. There are only few commercial dielectric polymer materials that meet the requirements: poly(methylmethacrylate) (PMMA) [42], polyvinylphenol (PVP) [43], amorphous fluoropolymer (CYTOP^®^) [44] and poly-*p*-xylylene derivatives (parylenes). Because of its unique properties described above, the latter polymer has the potential to outgrow the remaining candidates in its application as a gate dielectric in both single-crystal organic transistors and polycrystalline TFTs.

One of the most cited publications in the field of organic field effect transistors is the work of Podzorov et al. describing rubrene single-crystal transistors with Parylene C used as the gate insulating material [2]. This configuration allowed the authors to fabricate OFET devices with high charge-carrier mobility and reproducible characteristics. Parylene C forms transparent, pinhole-free conformal coatings of thicknesses as low as 0.1 μm with excellent dielectric and mechanical properties. Increasing thickness to 0.2 mm suffices to uniformly cover rough colloidal-graphite contacts. Transistors with rubrene as semiconductor and parylene as dielectric exhibit hole-type conductivity with a field-effect mobility up to 1 cm^2^/V·s and an on/off ratio up to 10^4^ at room temperature. Such a good performance has been made possible because of the high quality of both the rubrene crystals and the rubrene/parylene interface. The above results clearly demonstrate how important it is to select a compatible dielectric material with reduced number of charge traps [2].

The effect of the gate dielectric material on charge-carrier transport in single-crystal transistors was also discussed in the case of devices based on TTF derivatives, also characterized as a hole-type semiconductor. In this case, a more effective charge transport was observed when a Parylene C dielectric film was used instead of the Si/SiO_2_ combination. An application of parylene insulator also facilitated an evaluation of the influence that the crystalline structure of the semiconductor has on the performance of the single-crystal transistor. Of two crystalline forms of dithiophene-tetrathiafulvalene, the monoclinic alpha polymorph substantially outperformed the hexagonal beta polymorph [45].

The influence of the surface roughness of a dielectric film on the molecular arrangement of the first few semiconductor layers as well as on the size of its crystal grains is a critical factor in high-performance OFET devices. The effect of Parylene C roughness on charge transport has been studied in detail by an observation of submonolayer percolation of a pentacene film during its deposition on a rough dielectric surface. Interestingly enough, electrical properties of such a structure stabilize at the same film thickness as it does when a smooth substrate is used [46]. This suggests that the device performance will not be impaired by surface roughness as long as a conformal deposition of the semiconductor layer is guaranteed. These results are in agreement with the reports presented for a series of transistors with silicon/silicon dioxide substrates of various surface roughness [47–49]. It was found, that charge-carrier transport in relatively thick (multilayer) semiconducting films, obtained by thermal evaporation [47] or from solution [48] is insensitive to the substrate roughness. However, in thin monolayer semiconductor films the surface roughness significantly influences the charge-carrier transport [49]. This is due to the fact that charge-carrier transport in the initial monolayers is directly related to the roughness of a dielectric layer. Thicker films present lower sensitivity to the changes of surface roughness because each next layer away from the dielectric surface contains less and less defects. An increase of the domain size in the upper layers provides sufficient paths for charge carrier transport [48].

**Figure 8:**
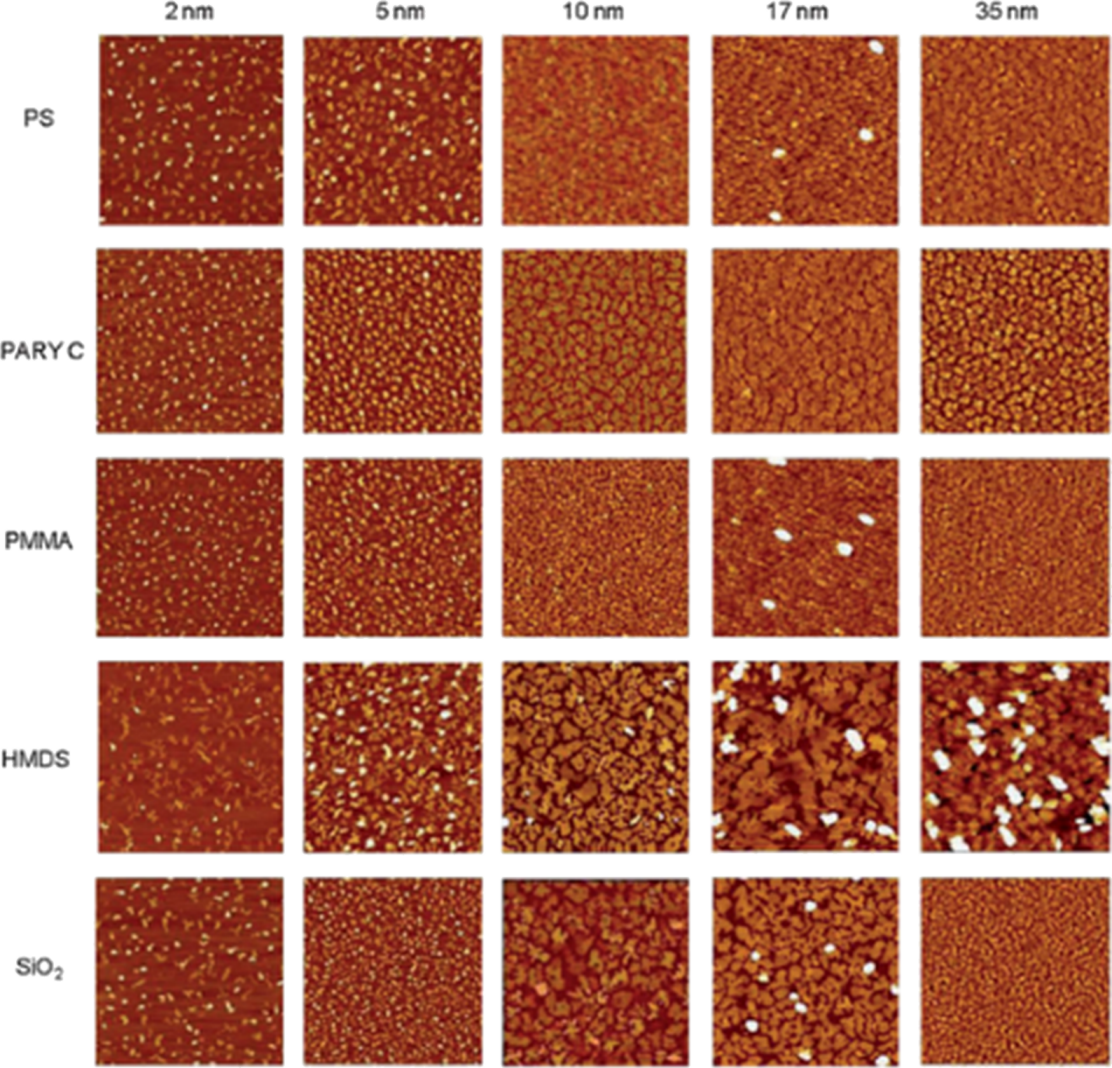
10 μm × 10 μm AFM images of tetracene thin films on different dielectric surfaces at different nominal thickness. *Z*-scale: 50 nm. Reprinted with permission from [50], copyright 2013 Royal Society of Chemistry.

Roughness is not the only surface parameter that may influence the supramolecular organization of the semiconductor film. The correlation between surface energy and charge transport in organic semiconductors has been discussed for TTF-based transistors produced on two different silicon dioxide substrates, characterized by surface energies of 51.8 and 40.1 mN/m, respectively [10]. It was found, that the average charge-carrier mobility was considerably higher (μ = 0.2 cm^2^/V·s ) when the SiO_2_ surface energy was lower. The substrate with the higher surface energy exhibited a mobility of μ = 0.006 cm^2^/V·s. More detailed studies were carried out for tetracene semiconductor films deposited on various dielectric materials, namely organic polystyrene (PS), Parylene C, and poly(methyl methacrylate) (PMMA) as well as on inorganic SiO_2_, with and without HMDS modification [50]. AFM measurements of tetracene semiconductor films show that the regularly shaped islands on the polymer dielectrics (PS, Parylene C, PMMA) lead to a complete substrate coverage at low nominal thickness, between 10 and 17 nm (Figure 8). Interconnected islands were formed at thicknesses of 10 nm and 17 nm, respectively, for PS and Parylene C films. This was enough to attain efficient charge transport in the tetracene layer. Certain differences in charge-carrier mobility and threshold voltages between PS and Parylene C were, however, observed.

The slightly lower charge-carrier mobility obtained for Parylene C has been attributed to the surface roughness, which increases the nucleation density and leads to less ordered films. The lower film order of Parylene C is compensated by the low charge trapping at the semiconductor/dielectric interface [50] confirmed by *I*_DS_ hysteresis observed for all devices, except those equipped with Parylene C.

**Figure 9:**
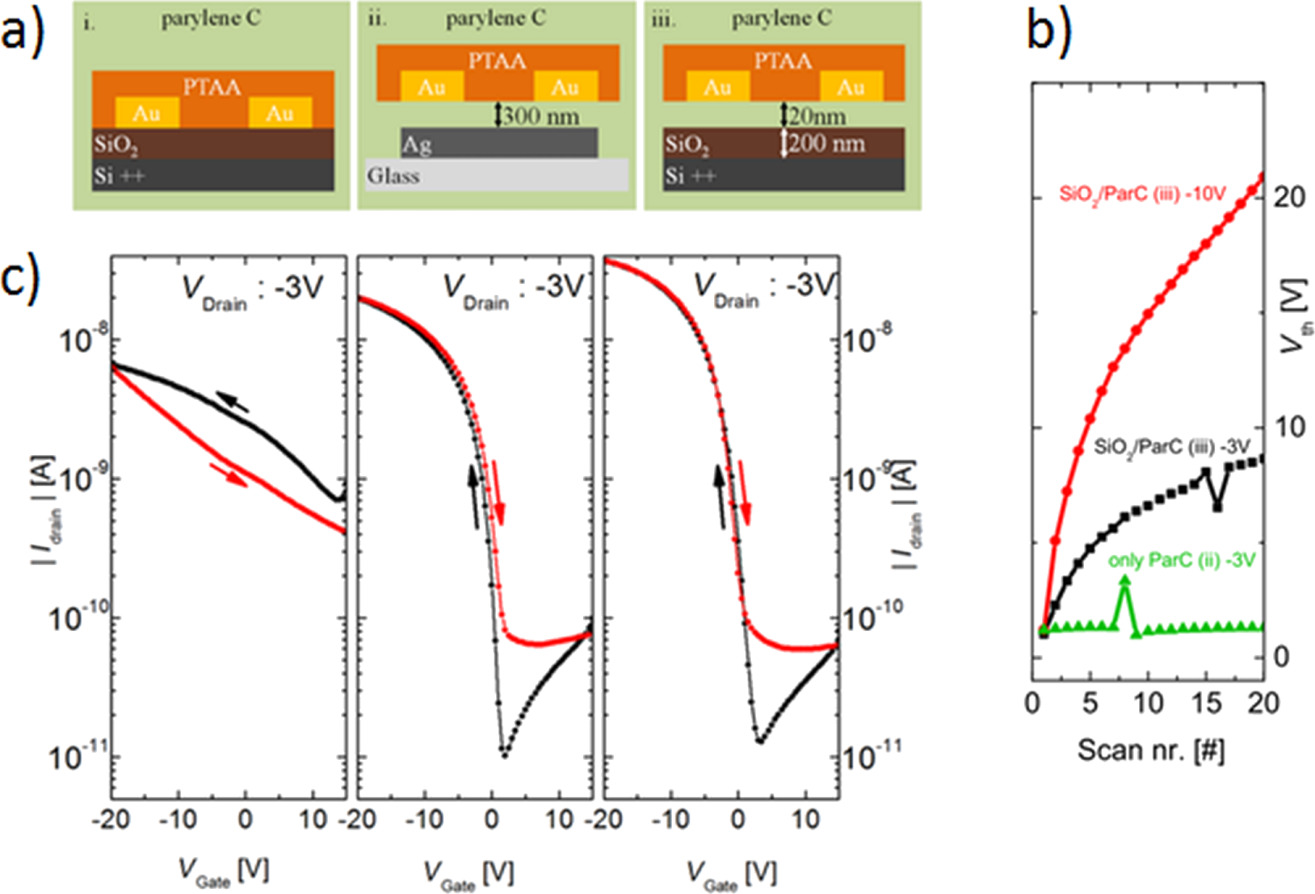
(a) Transistor architecture of the three different transistor stacks investigated, (b) threshold voltage trends of successive transfer sweeps for different *V*_D_, (c) representative transfer *I*–*V* characteristics of the three transistor stacks. The arrows in (c) indicate the sweeping direction of *V*_G_. Reprinted with permission from [51], copyright 2016 Springer.

The weak charge-trapping effect in OFETs with Parylene C dielectric in contrast to the SiO_2_ dielectric layer has been well demonstrated in the case of transistors based on poly[bis(4-phenyl)(2,5,6-trimethylphenyl)amine] (PTAA) [51]. The trapping significantly slows down the charge transport when SiO_2_ is used. By contrast, the PTAA transistor exhibits a marginal hysteresis between forward and backward sweep with similar transistor performance when the Parylene C is used either as self-standing dielectric (Figure 9a(ii)) instead of the SiO_2_ layer (Figure 9a(i)), or as a passivation layer (Figure 9a(iii)). In the latter case, the passivation effect is accomplished by Parylene C film creating a diffusion barrier that separates the conductive channel from electronic trap states in the SiO_2_ dielectric.

It is worth noting that the charge-trapping effect is not only connected to the dielectric/semiconductor interface. The effect of grain size and interface dependence of bias stress stability has been studied for C_60_-fullerene-based, n-type OFETs. It was revealed that, with an increasing grain size of C_60_, the bias stress induced shift of the threshold voltage can be controlled. This effect is mainly attributed to the mechanism of charge trapping at grain boundaries [52]. It was also found in further studies that the growth of C_60_ on the surface of Parylene C at elevated substrate temperatures leads to the creation of radicals at the interface between the active layer and the gate dielectric. The radicals formed during the C_60_ deposition help to improve the bias stress stability of C_60_-based n-type OFETs [53]. The creation of free radicals was also observed for a double-gate configuration with Parylene C as a dielectric layer [54]. This effect was not observed for the OFETs with top-gate configuration, when the Parylene C film was deposited on a top of the C_60_ layer.

**Figure 10:**
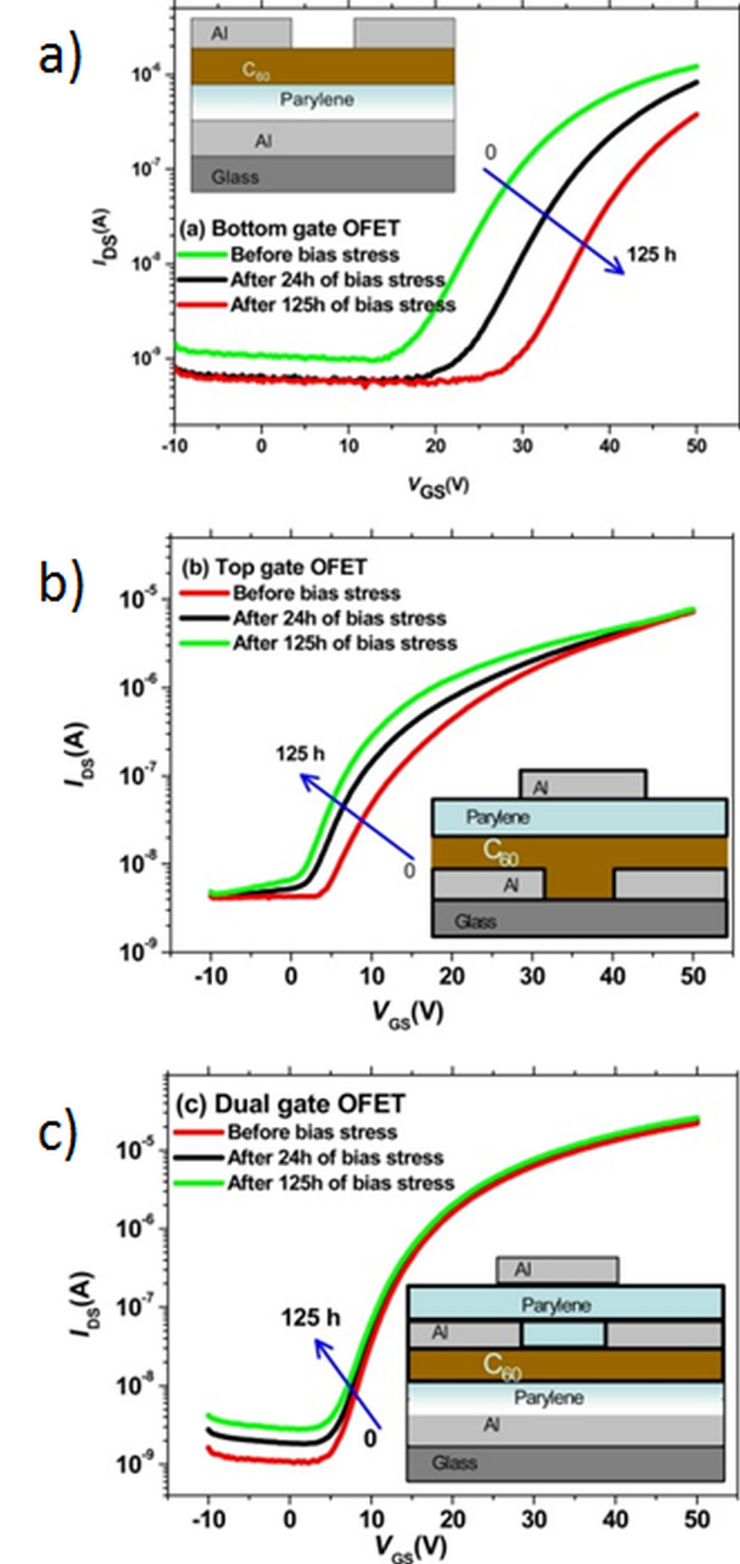
Transfer characteristics measured during the continuous bias stress of 125 h. (a) Bottom-gate, top-contacts, (b) top-gate, bottom-contacts, and (c) dual-gate OFETs. Reprinted from [54], copyright 2014 American Chemical Society.

As it was mentioned in the previous section, one of the major advantages of Parylene C films is the fact that they are deposited in a very clean environment, with no solvents and no initiators involved. This is a crucial point during the fabrication of the transistors with top-gate configuration where Parylene C is applied together with highly soluble n-type semiconductors as active material. There is a double advantage of such a combination: First, deposition of Parylene C by CVD method does not disturb the semiconductor surface, and second, the charge-trapping effect caused by oxygen and water is much less pronounced when Parylene C is working as a protecting layer of the semiconductor film. An example of this advantage is given in the abovementioned work with C_60_ fullerene transistors, where a comparison between the bottom-gate, top-gate and double-gate configuration with Parylene C as a dielectric layer is made [54]. The results are shown in Figure 10.

The charge-carrier field-effect mobility for bottom-gate, top-gate, and dual-gate OFETs was determined to be 0.1, 0.2, and 0.9 cm^2^·V^−1^·s^−1^, respectively. An application of the top-gate or dual-gate configuration not only increases the mobility value but it also brings about a different response to the bias stress. Figure 10 presents the transfer characteristics of the devices recorded before applying bias stress, and after 24 h and 125 h of bias stress application. While in the case of bottom-gate OFETs the *V*_th_ value is shifted towards more positive voltage (from 20.7 to 34.6 V), for the top-gate OFET configuration the bias stress results in a *V*_th_ shift in the opposite direction (from 14.3 to 0.1 V). In the case of dual-gate OFETs, only a small shift of *V*_th_ (from 11.5 to 8.5 V) was observed. The reason for this behavior is the fact that Parylene can chemically interact with C_60_ when it is being evaporated on top of the C_60_ semiconductor layer. A shift of *V*_th_ towards negative *V*_GS_ values implies an accumulation of metastable positive charges at the dielectric/semiconductor interface during the bias stressing. A similar behavior of bidirectional *V*_th_ shift was also observed in pentacene OFETs on silicon dioxide substrates modified by polydimethylsiloxane and it was assigned to either hole or electron trapping, depending on the bias stress polarity [55].

The top-gate configuration has also an additional advantage of the dielectric film working as a protective layer. In one example, a thin ordered layer of naphthalene bisimide was deposited via a zone-casting solution procedure with Parylene C used as the top-gate dielectric. Parylene was selected as a suitable material because it inflicted no damage to the semiconductor structure, a notion confirmed by relatively high charge carrier mobility of 0.18 cm^2^/V·s with accompanying threshold voltage below 5 V [24]. As it has already been mentioned, Parylene C plays a role of a protective layer (not only from mechanical point of view) for this *n-*type material for which the LUMO level of −3.77 eV does not assure stability under ambient processing conditions [56]. Interestingly, as it has been established in the course of device manufacturing, OFET parameters such as threshold voltage and charge-carrier mobility of n-channel transistors substantially depend on the material of the dielectric layer. Parylene C is superior for that purpose compared to fluorinated CYTOP polymer [57]. Manufactured via solution processing and equipped with Parylene gate dielectric, OFETs of adequate transport characteristics are operated under ambient conditions with no need of any extra shielding. After an initial period of a decrease of charge-carrier mobility, the long-term performance stabilizes at a satisfactory operational level.

It has to be stressed, however, that not all organic semiconductors show an increase of the charge-carrier mobility when the top-gate configuration is applied. For example, top-gate transistors with solution-processed dibenzo[*d*,*d*]thieno[3,2-*b*;4,5-*b*’]dithiophene semiconductor exhibit much lower mobility (0.0001 cm^2^/Vs) than a bottom-gate configuration (0.02 cm^2^/Vs) [58]. Changes in the surface energy between Parylene C (bottom gate, top contacts) and glass with gold electrodes (top gate, bottom contacts) are the main factor responsible for variations in the organization of semiconductor molecules. Additionally, parameters such as wettability and the corrugated surface can significantly alter the microstructure of semiconducting films and bring about a decrease of the device performance [18]. The effect of surface energy on charge-carrier mobility was discussed above using an example of transistors made of tetrathiafulvalene (TTF) derivatives on silicon dioxide substrates [10].

### Parylene C as an encapsulation layer

The origin of electrical instabilities of organic electronic devices is related to absorption of oxygen and/or water by the semiconductor film and to charge trapping in the semiconductor or at the dielectric/semiconductor interface. An efficient encapsulation should protect the organic semiconductor from interactions with gas and moisture and other adverse environmental conditions. Parylene C is one of the encapsulation materials that meet the above requirements [59–60]. However, there is certain ambiguity concerning the adhesion of this polymer to different substrates. According to the literature Parylene C exhibits a satisfactory adherence to gold, platinum and silicon nitride [61], which is, however, in contradiction to older reports [62–63]. Its adherence to polyimide, on the other hand, is found to be very low [61]. It appears that adhesion forces of Parylene C not only depend on the type of substrate, but they can also be easily modified by surface processing, such as oxygen plasma treatment or thermal annealing [64]. Which procedure is to be applied strongly depends on the material used and on the further application of the parylene layer. It should also be pointed out, that when Parylene C is applied as a flexible substrate, its limited adhesion to the temporary rigid support (used in the fabrication process as sacrificial material) constitutes a major advantage of this polymer.

The fact that Parylene C is sensitive to high-temperature treatment, such as thermal annealing, has been discussed in one of the previous chapters. When heated, this material becomes harder, more rigid and more brittle. A simple explanation of this effect is the increase of the degree of polymer crystallinity at elevated temperatures. In a similar way, when deposited at higher pressure, Parylene C layers are more elastic and less brittle because of lower crystallinity. Taking the above consideration into account, care should be taken not to operate at exceedingly high temperatures, which is a likely limitation of the entire field of organic electronics.

One of the early applications of Parylene C encapsulation layer in an electronic structure was that of a microelectrode insulator [65]. The Parylene C-covered iridium and tungsten microelectrodes were investigated by means of in vivo and in vitro impedance tests. In vitro studies were carried out in an especially prepared chamber containing saline, either sterile or plasma-incubated at 37 °C, in order to reproduce the natural environmental. In vivo testing was performed by an implementation of multiple electrode systems in monkey motor cortex [65]. In these studies, an unchanged impedance of the microelectrodes protected by Parylene C layers has been recorded for over four months [66]. As another positive result, no destructive influence of the encapsulation material was observed when Parylene C had been employed to protect a pentacene OFET device, where no remarkable alteration of the current–voltage characteristics before and after an application of a passivation layer was recorded [67]. Because of the specific properties of the parylene deposition procedure taking place at room temperature, no changes in the semiconducting channel were induced and the device fabricated showed unchanged transfer and output characteristics.

**Figure 11:**
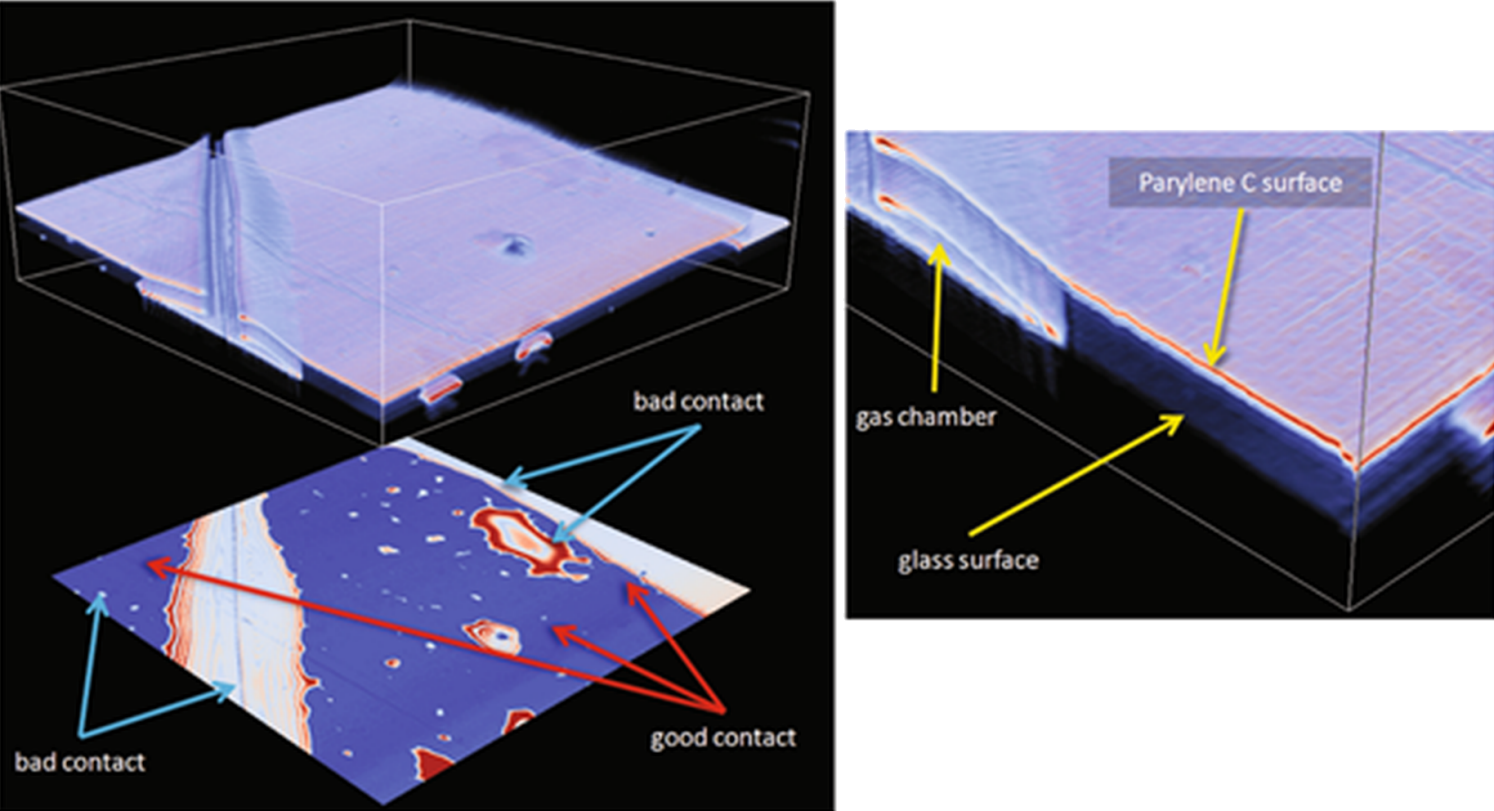
Volumetric reconstruction of the Parylene C-coated microscopy glass (left, atop) and calculated amplitude map of the Parylene C/glass interface (left, bottom). Boundary box indicates the size of the volume 2000 × 2000 × 208 µm. Zoom-in image (right). Coating defects and gas chambers are clearly visible. Reprinted with the permission from [68], copyright 2011 Springer.

**Figure 12:**
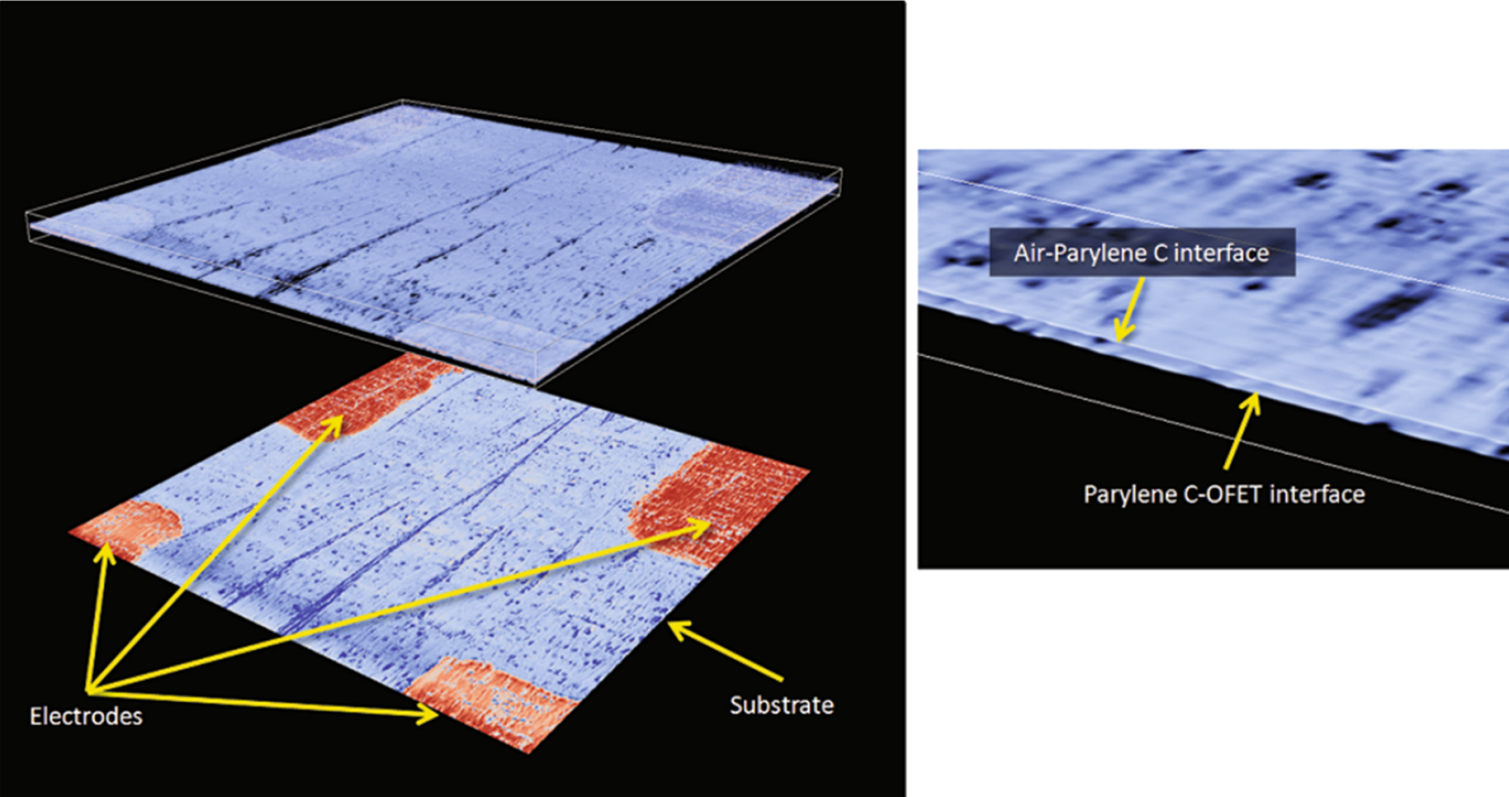
Volumetric reconstruction of the Parylene C-coated OFET structure (left, atop) and calculated amplitude map of the Parylene C/substrate interface (left, bottom). Boundary box indicates the size of the volume 2000 × 2000 × 73 µm. Zoom-in image showing interfaces of 2 µm thin polymer layer (right). Reprinted with the permission from [68], copyright 2011 Springer.

The quality of thin protective films of Parylene C was investigated by optical coherence microscopy (OCT), whereby defects in the encapsulation layer were detected, either by a change of the number of peaks in the interference fringe signal envelope, or as a change in the signal amplitude [68]. Figure 11 presents a glass substrate covered with 1 μm thick Parylene C film, with gas chamber and bad contact areas purposefully created as a reference sample for further investigation of transistors. Transistors with the typical bottom-gate, top-contact configuration and with Parylene C used as the encapsulation layer were investigated. Figure 12 presents a volumetric reconstruction of Parylene C-coated OFET as well as a calculated amplitude map of the Parylene C/semiconductor interface, where zoom-in image (right panel) shows the interface without defects. To summarize, it can be concluded that by optimizing the process of Parylene C deposition no defects in the semiconductor layer and at semiconductor/encapsulation layer interface are formed and, therefore, no additional charge traps are created at that interface.

**Figure 13:**
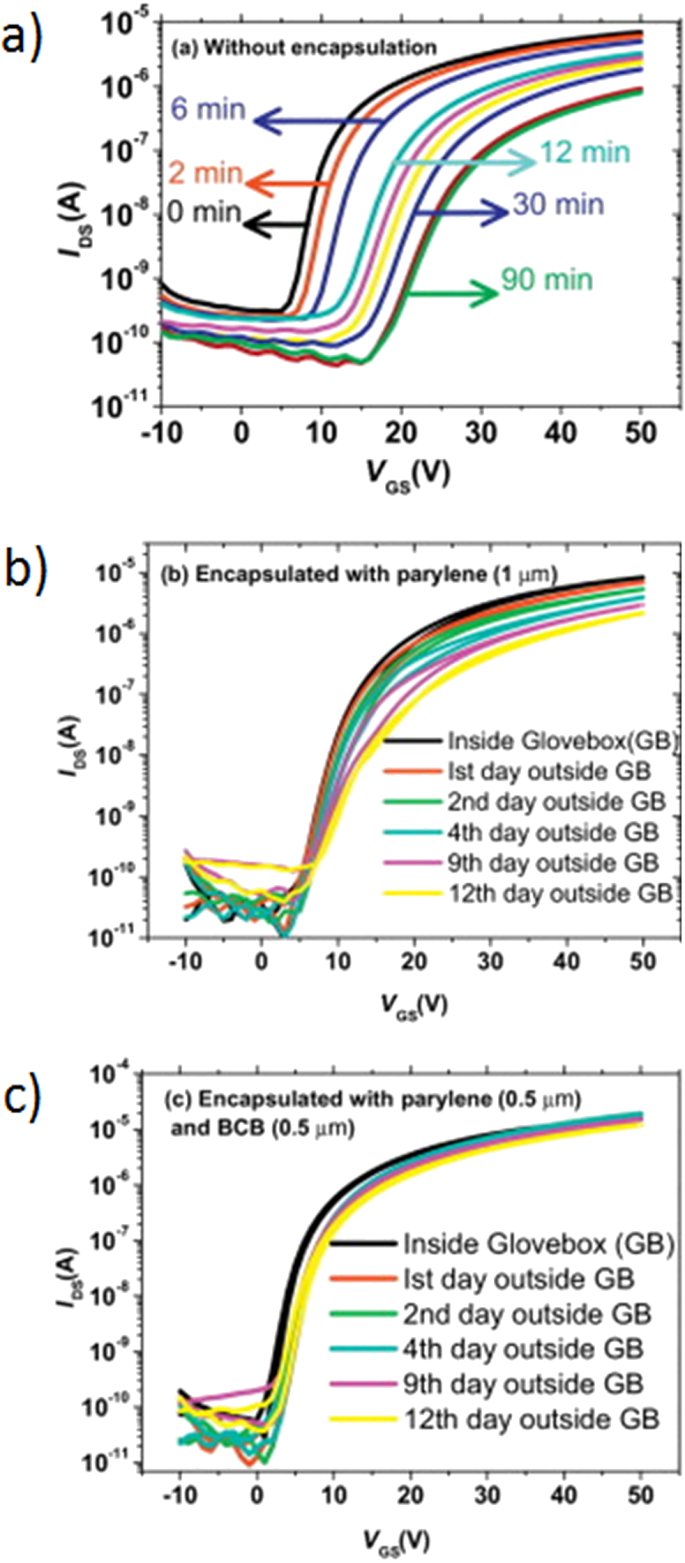
Transfer characteristics recorded under ambient conditions of a fullerene transistor without encapsulation (a), encapsulated with 1 μm thick layer of Parylene C (b) and encapsulated with 0.5 μm thick layer of Parylene C followed by 0.5 μm layer of benzocyclobutene (c). Reprinted with the permission from [69], copyright 2014 Elsevier.

It should be pointed out that the results discussed above were obtained for active materials that were not sensitive to ambient conditions. However, most *n-*type organic semiconductors do not show such stability. One example of an unstable material (characterized by considerable charge trapping) is the previously described fullerene C_60_. The transistor characteristics of unprotected and Parylene C protected fullerene based devices are presented in Figure 13.

Transfer characteristics, monitored under ambient conditions, of non-protected OFETs are shown in Figure 13a. As seen in the figure, the source–drain current (*I*_DS_) of a non-encapsulated OFET decays over 90 min by one order of magnitude, with the gate threshold voltage shifting to higher magnitudes [69]. This behavior strongly indicates that oxygen and/or water vapor create charge trap states the filling of which requires higher gate voltage for a successful OFET operation. The transfer characteristics of OFETs equipped with a protective layer of a sole 1 μm thick Parylene C coating and a combination of 0.5 μm thick Parylene C with 0.5 μm thick benzocyclobutene (BCB) films, monitored under ambient conditions for twelve days, are presented in Figure 13b and Figure 13c, respectively. The encapsulation layer of Parylene C substantially improves the air stability of the C_60_-based n-type OFET. In this case, the decay of *I*_DS_ current of one order of magnitude has been recorded after 12 days. The onset voltage remains the same but a small shift in the threshold voltage is observed [69]. The slow degradation of *I*_DS_, measured in the OFET encapsulated with Parylene C (1 μm) may be attributed to the slow penetration of water vapor and oxygen through the encapsulation layer. The decrease in the permeability of water vapor and oxygen through the bilayer encapsulation film has been attributed to the sealing of grain boundaries by the smoothness of the BCB layer. However, it only works when Parylene C/BCB bilayer system is used. By applying a bilayer encapsulation system, the defects in the Parylene C film underneath are blocked by the BCB layer. The permeation path for water vapor and oxygen becomes tortuous, which results in an improvement of the barrier performance.

Finally, it is also worth to add, that when Parylene C is used as a gate insulator in OFET transistors with top-gate configuration, its protective properties are considerably enhanced by a metal gate electrode deposited on its top. This feature has been found especially useful in the case of OFETs equipped with either ambipolar [28] or *n*-type [24] channels, since the *n*-type organic semiconductors are particularly sensitive to a deteriorative effect of atmospheric oxygen and water vapor.

## Conclusion

The presented review of literature describing state-of-the-art applications of Parylene C as substrate, dielectric, insulator or protecting and encapsulating material in construction of OFETs demonstrates that poly(*p*-xylylenes) constitute a class of versatile supporting materials particularly suitable for applications in flexible organic electronics. The properties of greatest importance for such applications are the extraordinary purity and chemical inertness of Parylene layer, its elasticity and ability to form smooth and pinhole-free conformal coatings. Due to high purity and low dielectric permittivity, the concentration of charge-carrier traps at the Parylene/semiconductor interface is very low. This results in enhanced charge-carrier mobility in the OFETs. The flexibility of Parylene C paves the route for flexible electronics, and the continuous and conformal coating, when combined with metal gate electrodes evaporated on the top of parylene layer, assures a sufficient protection of OFETs against oxygen and water, which is especially important for transistors with *n*-type channels.
